# Group-Treatment for Dealing with the Work-Family Conflict for Healthcare Professionals

**DOI:** 10.3390/ijerph182111728

**Published:** 2021-11-08

**Authors:** Nicole Rosalinde Hander, Manuela Gulde, Thomas Klein, Nadine Mulfinger, Lucia Jerg-Bretzke, Ute Ziegenhain, Harald Gündel, Eva Rothermund

**Affiliations:** 1 Department of Psychosomatic Medicine and Psychotherapy, Ulm University Medical Center, 89081 Ulm, Germany; thomas.klein@uni-ulm.de (T.K.); nadine.mulfinger@gmx.de (N.M.); harald.guendel@uniklinik-ulm.de (H.G.); 2 Department of Child and Adolescent Psychiatry/Psychotherapy, Ulm University Medical Center, 89075 Ulm, Germany; manuela.gulde@uniklinik-ulm.de (M.G.); ute.ziegenhain@uniklinik-ulm.de (U.Z.); 3 Department of Psychosomatic Medicine and Psychotherapy, Medical Psychology, Ulm University Medical Centre, 89075 Ulm, Germany; lucia.bretzke@uni-ulm.de

**Keywords:** work-family conflict, workplace intervention, healthcare workers, children’s well-being, self-efficacy

## Abstract

Healthcare professionals’ exposure to work-family conflict negatively affects the health and well-being of the whole family and organizational outcomes. Specified workplace interventions are lacking. Therefore, the aim of the study was to evaluate the feasibility of a two-day group-treatment specifically designed for the needs of healthcare professionals with family responsibilities concerning participation, satisfaction with the intervention and family- and individual-related outcome variables. 24 mostly female (85.7%) participants of a community hospital in southern Germany attended the treatment. Data were collected at baseline (T0), directly after the treatment (T1) and two months later (T2). A two-factor analysis of variance with repeated measures showed a statistically significant time x group effect for self-efficacy (*F* = 5.29, *p* = 0.011). Contrasts displayed substantial pre-post (T1-T0, T2-T0) increases of self-efficacy in the intervention group as compared with the control group. Non-parametric Mann-Whitney-*U* tests are in line with these findings. The results indicate that the group-treatment adapted to the needs of healthcare professionals has the potential to boost self-efficacy among healthcare professionals and that participants were predominantly satisfied. Perspectives for future research and practical implications are discussed in the light of the manifest lack of healthcare professionals.

## 1. Introduction

“Balance” of work and family lives is a much sought-after but a hardly claimed state of being. Conflict between work and family life (work-family conflict) has been commonly defined as a form of inter-role conflict in which the demands of the role from both domains are mutually incompatible in some respect [1].

In the healthcare sector, an alarming situation is emphasized in this domain, as healthcare professionals are confronted with specific work-related demands like shift work, long working hours, unpredictable changes of the work schedule, high quantitative workload, working under pressure, and high emotional commitment [2,3,4,5,6,7,8,9,10,11]. Furthermore, the lack of healthcare professionals due to demographic change and the foreseeable shortage of skilled workers in Germany and other countries might increase work-related demands [12,13,14,15,16,17,18,19,20,21,22].

Work-family conflict (WFC) is considered as a predictor of health determinants, well-being, and organizational outcomes [3,4,23,24,25,26,27,28,29,30,31,32,33,34,35,36]. Findings in Germany show that WFC is correlated with personal burnout and behavioral and cognitive stress symptoms [4,34] and poor health [28,37]. For example, double-duty caregivers providing informal care to a family member in need are at high risk of developing symptoms of overload [38,39]. Furthermore, WFC was found to be negatively associated with parental self-efficacy and the perceived quality of parent-child interaction [40]. Parents’ WFC constitutes a potential determinant of children’s health [41,42,43,44] and subsequently a target for prevention and health promotion [45,46]. With 50 percent of nurses reporting chronic work interference with family [47], WFC is considered as one of the main reasons to quit the nursing profession [9,48]. WFC is also highly prevalent among German hospital physicians [4,7,49], and it is strongly related to the intention to leave the job or to go abroad [4]. WFC was found to be particularly relevant for female physicians’ intention to leave direct patient care [15]. The WFC situation becomes even more critical as female physicians form an increasingly large part of the physician workforce [50]. As a consequence, WFC plays a critical role for the choice of the place of employment among German physicians [51].

Therefore, measures and interventions in the workplace are urgently needed in order to reduce demands and to increase resources [52]. Flexible working hours, in-service training during parental leave and substitution on short call, among other options, have been discussed in Germany [53]. But organizational and political initiatives have not proven able to deal with these issues sufficiently so far [54,55]. One further starting point for treatment could focus on encouraging the individual psychologically in the daily struggle between work and family life. Individual resources such as self-efficacy might prevent WFC [7,56,57]. Self-efficacy is defined as the modifiable, personal agency and belief of being able to control challenging environmental demands by means of taking adaptive action [58,59]. Personal resources like self-efficacy were supposed to mediate the relationship between demands and detrimental outcomes [7,60,61]. They are related to performance and job satisfaction [62]. The individuals’ work-family specific self-efficacy predicted their perceptions of future work-family conflicts [63]. As reported by Smoktunowicz and colleagues [64], demands associated with increased work-family conflict decreased self-efficacy and resulted in heightened stress at work and in family. Recent research suggests that cognitive-behavioral therapy-based stress management programs are successful at enhancing participants’ perceived self-efficacy [65]. Further evidence was found for a parenting intervention aimed at improving occupational self-efficacy [66].

For the healthcare sector, research investigating how to improve coping with WFC is generally rare [67]. Evidence indicates that cognitive-behavioral therapy interventions and mental and physical relaxation are effective at reducing occupational stress in healthcare workers [68,69,70]. However, treatments focusing on stressors specific to WFC by taking the healthcare worker’s as well as the child’s needs into account are lacking, for example how to arrange divergent demands. Therefore, we developed a sustained group-treatment based on the Intervention Mapping Approach (IMA) by Bartholomew [71] within the research association “Mental Health at the Hospital Workplace” (SEEGEN).

This is the first experimental trial to evaluate a group-treatment focusing specifically on the interplay between work and family lives for healthcare professionals. The aim of the study was to investigate the feasibility in participation, satisfaction with the group-treatment, and effects on the outcome measures of self-efficacy, work-life balance and the participants’ children’s health and well-being.

## 2. Materials and Methods

### 2.1. Design

The two-arm quasi-experimental study design compared the two-day group-treatment for coping with the work-family conflict in an intervention group (IG) with a waiting control group (CG) that received treatment as usual (TAU). Participants in the CG didn’t receive the group-treatment before completion of data collection. Due to eligible participants’ professional obligations and related hindered recruitment, the initially planned RCT was converted into a quasi-experimental trial [72,73]. The quasi-experimental design allows for allocation either to the IG or the CG according to participants’ chosen preferable date of treatment.

### 2.2. Sample

Potentially eligible participants were all 1270 adult employees of a community hospital in southern Germany regardless of gender in the functional, nursing, medical or other service. Participants were included if they had family responsibilities, including expecting a baby or being a parent. Exclusion criteria were a lack of sufficient German language skills.

### 2.3. Procedures

Through posters and announcements of supervisors, hospital employees were invited to information events (offered once in November 2017, twice in December 2017, and once in April 2018). Whereas participants and data collectors were kept blinded to the allocation according to the participants chosen preferable date of treatment, the trainer was aware of participants’ allocated study arm. Outcome parameters were assessed with questionnaires at baseline (T0; two to four weeks before the start of the treatment), immediately after the treatment (T1), and two months later (T2). At T0 and T2, the paper-pencil questionnaires were posted to participants’ indicated addresses, and they sent it back in a specific envelope to the researchers. At T1, participants received the questionnaires in person. Participants were encouraged to answer the questions spontaneously, as much as possible, without being disturbed. Data were gathered between 1 May and 30 September 2018. The CG was offered the intervention four months later than the IG after having completed the questionnaires at T2. In each study arm, two workshops were provided with a maximum of 12 participants in each.

### 2.4. Intervention

A specifically tailored group-oriented seminar (nine teaching units lasting 60 min each, over two consecutive days) was conducted by an experienced occupational health professional trainer. The program is proposed for parents with children from 0 to 18 years. All trainings took place in seminar rooms located at the hospital. Rooms were equipped with a computer, a projector, and yoga mats, and provided sufficient space for the group. Participants were seated in a semicircle facing the projector and each other. During group work, groups of two to three participants were formed. Practical stress management in the form of an Ashtanga yoga class was facilitated by an experienced yoga teacher. Participants used the provided yoga mats for this purpose. The group-intervention took place during participants’ regular working time, from 9:00 a.m. to 4.30 p.m. the first day and from 9:00 a.m. to 1:00 p.m. the following day. All participants were asked to verbally give a confidentiality agreement.

The training was specifically designed to foster awareness of the individual work-family conflict, to identify individual resources and obstacles, to strengthen individual resources and to deal with obstacles, to transfer knowledge concerning the work-family conflict and to empower individuals to practice appropriate self-management techniques. The approach combines the underlying principles of behavioral therapy techniques [74] (e.g., psychoeducation, behavior building, modification, and attitude change), emotion-centered principles (e.g., focus on feelings concerning the work-family conflict), and group dynamic factors [75] (e.g., vicarious learning) which provide impulses for reflection of attitudes and self-efficacy expectations. In addition, the workshop was intended to help sharing experiences, acknowledging the difficulties of others, and fostering group cohesion.

Based on a qualitative needs assessment, and a detailed literature research [76,77,78,79] according to Bartholomew and colleagues [80], modules focusing on family, job and stress with lectures, analysis of resources and inhibiting factors and the development of next steps were created. A manual with worksheets was developed in an interdisciplinary team to standardize the pretested program. The manual is in preparation for publication.

After address of welcome and a short introduction, participants’ personal expectancies and aims were explored. Afterwards, contents and aims of the group-treatment were presented. Participants were expected to analyse their individual work-family conflict in individual and group work and share their own experiences in the safe group space. With the help of worksheets, participants were encouraged to reflect upon their resources, for instance, how to activate individual valued resources and how these could offset given demands and inhibiting factors concerning family as well as job life. After initial individual and group work, the group searched for the best possible solutions with the help of the experienced trainer. To foster self-management, the development of a next step of action was encouraged. Further elements were lectures regarding the connection between stress experience, stress response and the influence of personal stress on the relationship with the child as well as time management and organization. The analysis of individual stress symptoms and strategies to better deal with stress due to work-family conflict was guided by worksheets. Group exchange was encouraged by the experienced trainer. In addition, lectures on developmental psychological findings and attachment theory for practical everyday life as a parent were developed. These included the developmental needs of children and parenting-related issues (transition to institutional/extra-familial care, “what is normal”, “when are children stressed out”), partnership and changes in the transition to parenthood. Lectures via PowerPoint presentations were aimed at keeping parents informed, validating their individual situation and, at the same time fostering participants’ awareness of their own (dysfunctional) patterns in stressful situations and respective consequences regarding the health of the child. The practicing of self-guided interventions was encouraged instead, such as abdominal breathing, self-distancing from dysfunctional thoughts and unpleasant feelings, and regular self-care, especially with regard to creating social bonds. The trainer inspired participants to practice individually appropriate self-care by presenting several tools. For example, the participants were instructed on practical stress management in the form of Ashtanga yoga. Additionally, a 10- and a 30-min yoga sequence were developed and provided as online movie clips in cooperation with a yoga institute. Specific attention was paid to a simple exercise sequence that could be learned quickly and could be easily integrated into work and family lives. In contrast to a pure coping workshop, this workshop also highlights dilemmas [81] in the context of role play to better deal with a difficult work-family conflict situation in a further step. At the end of the workshop, participants fixed/repeated the developed next steps of action under activation of personal resources. Furthermore, all participants were required to reflect upon which aspects of the workshop were most beneficial for them.

After the group-treatment, participants were expected to transfer their developed next steps of action and to practice regularly the 10- and the 30-min yoga sessions. Additionally, they were instructed to strengthen their theoretical understanding and keep personal analysis in mind by reading the manual and reflecting upon worksheets used during the group-treatment.

### 2.5. Measures

Socio-demographic data and answers to job- and family-specific questions were recorded at baseline.

Feasibility indicators were number of participants eligible, those willing to participate in the study, the number of participants lost to follow-up and satisfaction with the specific-tailored group-treatment.

Satisfaction with the group-intervention was assessed directly after the intervention with two self-constructed four item questionnaires and the opportunity to give written feedback in an open text format. One questionnaire asked (on a five-point Likert scale ranging from “not at all” to “a lot”) about general satisfaction (e.g., “Participation in the workshop was helpful with my everyday life”). The other instrument asked on a five-point Likert scale ranging from “bad” to “very good” about satisfaction with the modules (therapeutic input, practical exercises, yoga, and group exchange).

Occupational and family self-efficacy was assessed with the Adapted General Self-Efficacy Scale (AGSE) [82]; Table A1). The items were expanded by supplementary phrases for work-family compatibility (e.g., “For every problem concerning my job or my family, I can find a solution.”). The instrument consists of 10 items rated on a four-point Likert scale ranging from 1 (not at all true) to 4 (completely true) with higher sum scores reflecting higher occupational and family self-efficacy (range 10 to 40). Several investigations proved AGSE to be a reliable and valid instrument [83].

Work-life balance was assessed with the Trierer Scale to measure Work-Life Balance (TS-WLB; in German: Trierer Kurzskala zur Messung von Work-Life Balance (TKS-WLB) [84]). The construct refers to affective and cognitive perceptions of how well work and non-work roles fit together and are managed in accordance with life values, goals, and aspirations [85]. The instrument consists of five items (e.g., “I am satisfied with the balance I achieved between my work and family life”) rated on a five-point Likert scale ranging from 1 (strongly disagree) to 6 (strongly agree) with higher mean scores reflecting higher work-life balance. The TS-WLB captures individuals’ experiences in a set of equal-weighted personal roles and highlights the aspect of recovering from demands [84]. Support for the reliability and validity of the work-life balance scale is provided [84].

Children’s health related quality of life was measured with the KIDSSCREEN-10 index in the proxy measure for parents [86], with higher sum scores (range 10–50) reflecting better quality of life. The 10 items rated on a five-point Likert scale ranging from 1 (not at all/never) to 5 (extremely/always) address affective symptoms of depressed mood, cognitive symptoms of disturbed concentration, psycho-vegetative aspects of vitality, energy and feeling well, and psychosocial aspects correlated with mental health, such as the relationship with the their parents (e.g., “Has your child felt that his/her parent(s) treated him/her fairly?”). Good psychometric properties such as the ability to differentiate between different groups of mental/physical health were reported [86]. Regarding the proxy report, convergent validity with other children self-report health-related quality of life questionnaires for the KIDSCREEN-10 children self-report (*r* = 0.43 to *r* = 0.63) and to a lesser extent with the KIDSSCREEN-10 parent proxy report (*r* = 0.22 to *r* = 0.40) was shown [86].

### 2.6. Data Analysis

Feasibility indicators were reported descriptively.

Missing single items were replaced in each scale by the individual’s mean of the available values [87]. However, if the complete questionnaire was missing, the participant was excluded from the respective analysis according to the per-protocol (PP) analysis. The number of allowed missing values complied with the instructions of the test authors (at maximum 20% of missing values had been allowed) except for the KIDSSCREEN-10-Index with 20% of missing values allowed. The instrument was validated for children and adolescents aged from eight to 18 years, and we included parents with children of other age groups as well.

We used *t*-tests for normally distributed values, Mann-Whitney-*U* tests for variables that did not meet the assumption of the normal distribution, and chi-square tests for analyses of differences between the IG and the CG in baseline values of the target variables and demographic characteristics. Group differences in all target variables over time were investigated using a mixture of three-factor repeated measures (T0, T1, T2) and between-groups measures (IG, CG) ANOVA, a mixed design. AGSE was normally distributed for all groups except for T0 of the IG (*p* = 0.048) using the novel, as assessed by the Shapiro-Wilk test (*p* > 0.05). TS-WLB and KIDSSCREEN-10 index were normally distributed for all groups, as assessed by the Shapiro-Wilk test (*p* > 0.05). Differences from T0 to T1 and T0 to T2 (T1-T0, T2-T0, difference score > 0 indicating an increase in the respective outcome variable) were calculated by conducting contrast analyses. Due to the small sample size, we calculated both the parametric as well as the non-parametric Mann-Whitney-*U* test. 95% confidence intervals (95%-CI) were computed.

To appraise clinical relevance, effect sizes were calculated. We reported the amount of variance in follow-up outcomes that was explained by the intervention over and above the baseline values of outcomes (partial eta square, ɳ_p_^2^). In line with Cohen [88], we regarded ɳ_p_^2^ = 0.01 as small, ɳ_p_^2^ = 0.03 as medium and ɳ_p_^2^ = 0.14 as large effects. An approximate effect size, *r*, which is calculated from the *z*-score/from the *F*-ratio, was used for the non-parametric tests/contrasts with an effect size less than 0.1 as a small effect, between 0.1 and 0.3 as a medium effect and greater than 0.5 as a large effect [89]. All data management and statistical analyses were conducted using IBM SPSS statistics 25 (IBM Corporation, Armonk, NY, USA).

### 2.7. Ethical Approval and Registration

This study was conducted according to the guidelines of the Declaration of Helsinki and approved by the Ethics Committee of Ulm University (204/17; date of decision: 31 July 2017). The investigation was registered by the German Clinical Trial Register (DRKS) under ID number DRKS00013544.

## 3. Results

**Figure 1:**
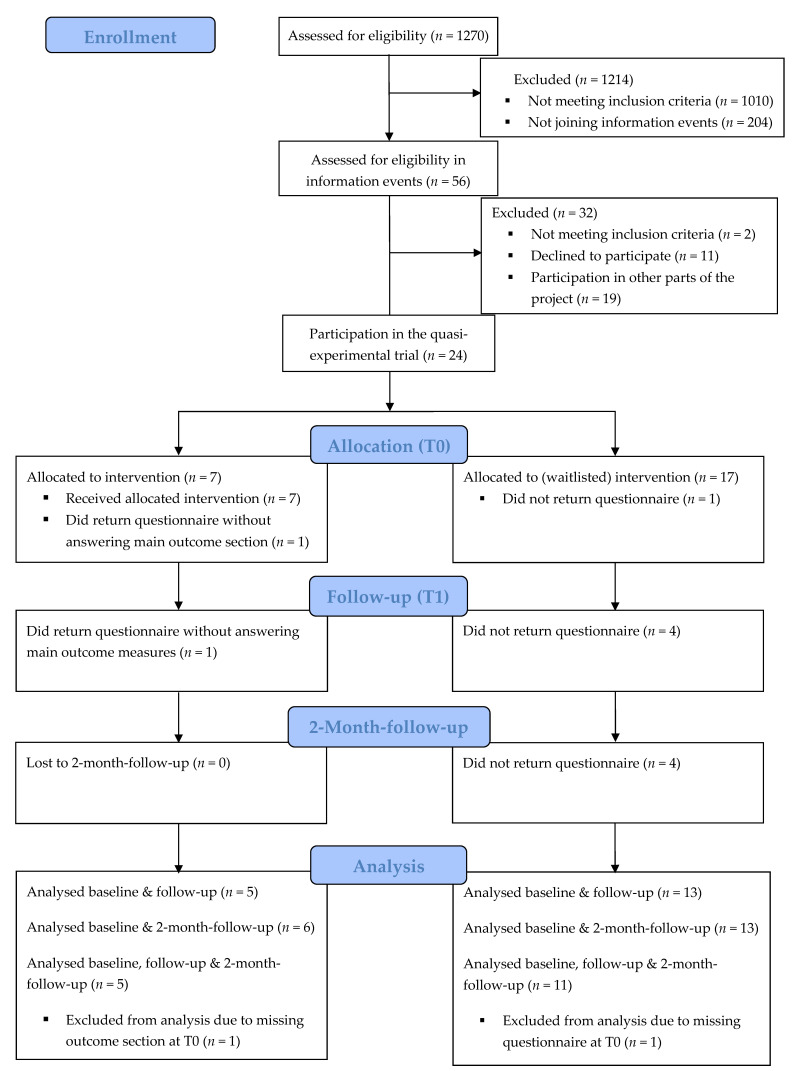
Selection process dataset. T0–T2 measurement points at baseline, directly after the group-treatment and two months later.

Of the 56 employees interested in study participation, 24 were included. The participant flow during the study according to the CONSORT statement [90] is presented in Figure 1. Recruitment was hindered due to participants’ professional obligations. Ultimately, 16 (66.7%) of the 24 participants completed the follow-up assessments. Reported reasons for the failure to follow-up were professional obligations. Those who did not accomplish all three assessments did not differ significantly from those who responded to all questionnaires regarding demographic characteristics and baseline levels of the proposed outcomes. Likewise, dropouts in the IG did not differ significantly from dropouts in the CG.

The demographics of our sample are displayed in Table 1. All participants had at least one child except for one person in the CG who did not give any information (4.2%). One person in the CG reported that no children lived in their joint household any longer (4.2%); two participants in the CG did not give any information (8.3%). At baseline, no significant differences between the IG and the CG in socio-demographic, professional and family characteristics were found which also holds for the outcome variables between the IG and the CG (Table 1) and those participants included in the respective 2 × 3 ANOVAs.

**Figure 2:**
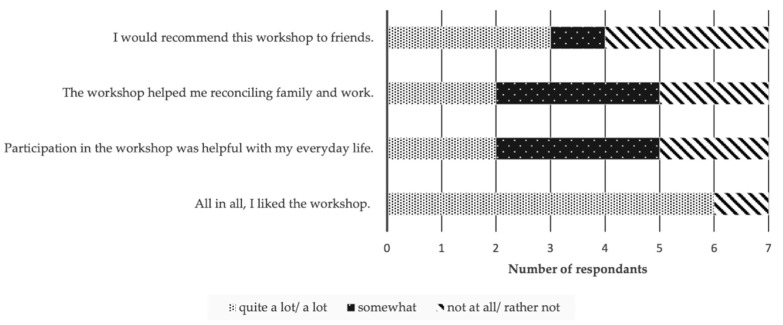
Satisfaction with the group-intervention.

**Figure 3:**
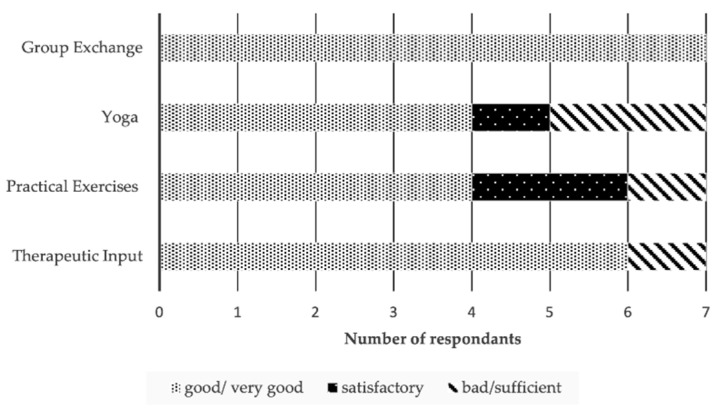
Satisfaction with parts of the group-intervention.

Findings about satisfaction with the group-treatment are shown in Figure 2 and Figure 3. Overall, participants showed high satisfaction (85.7%). For more than half, the workshop was helpful in coping with the work-family conflict. More than half would recommend it to a friend (Figure 2).

Especially group exchange and therapeutic input were regarded as relevant (Figure 3). In addition to open questions, trainers and peers received sample feedback from participants which is summarized in the text below. Participants highlighted the group interaction which was characterized by openness, trust, and respect. Some felt relieved when talking about their individual situation in the safe group space. Chosen topics were regarded as relevant. However, some felt the modules were too theoretical and wished to have more time for their own compatibility analysis. Others thought that a two-day workshop took up too much time. Some participants proposed the creation of several workshops or workgroups according to the age of their children. Others suggested highlighting the role of employers in supporting work-family balance.

Descriptive statistics, intercorrelations, and internal consistencies of all target variables at baseline, follow-up and two-month-follow-up for the total sample are displayed in Table 2. Cronbach’s alpha values indicate acceptable to excellent internal consistencies in all applied scales.

There were only a few missing single items in our sample that were handled according to the procedure described in the Section 2.6 Data Analysis.

The results of the two-factor analysis of variance with repeated measures for the outcomes of self-efficacy, work-life balance and children’s health related quality of life are shown in Table 3. Differences from T0 to T1 and T0 to T2 by conducting contrast analyses are presented in Table 4.

A two-factor analysis of variance with repeated measures for the outcome of self-efficacy showed a statistically significant time x group effect (*F* = 5.29, *p* = 0.011; Table 3). Contrasts indicated significant pre-post (T1-T0, T2-T0) increases of self-efficacy in the IG as compared with the CG, with large effect sizes (Table 4).

In terms of the outcome of work-life balance, there was a significant time x group effect (*F* = 8.53, *p* = 0.001; Table 3). A contrast indicated a significant reduction of work-life balance between T0 and T2 but not between T0 and T1 in the IG as compared with the CG (Table 4). 

There were no significant changes in children’s health related quality of life (*F* = 1.40, *p* = 0.267; Table 3). 

Non-parametric Mann-Whitney-*U* tests are in line with these findings except for the outcome work-life balance between T0 and T2 (*U* = 17.00, *z* = −1.94, *r* = 0.45, *p* = 0.058).

## 4. Discussion

This is the first study to evaluate the feasibility of a group-treatment focusing specifically on the interplay between work and family life for healthcare professionals and their youth. Our results indicate that most participants were satisfied with the intervention, and that it helped them to feel more competent about managing competing work and family demands.

Participants were predominantly satisfied (85.7%) with the treatment. All participants rated the group exchange as good or very good. This exchange may have helped many participants realize that they were not alone in their daily struggle with combining work and family life. According to Yalom [91], universality of suffering is one change factor in group psychotherapy. Some highlighted the fact that the conversation with members of different professional groups was enriching. This preliminary result is consistent with findings that showed that team health climate as a contextual resource facilitated health-related outcomes among employees [92], and that strong social and collegial support enhanced a nurse’s capacity to cope with work and personal stress [93]. The results might give a hint that employees’ shared perceptions of the extent to which their team is concerned, cares and communicates helps in building resilience within the workforce.

Difficulties concerning recruitment of hospital workers were supported by their participation in the study The low participation rate of hospital workers, as only 24 of 48 group-treatment places were taken by them, is in line with general difficulties concerning recruitment of hospital workers [55,94]. It has been repeatedly reported that hospital staff face hazards such as long and irregular working hours, physical burdens, understaffing, social or role conflicts and many more [55]. Barriers in the recruitment of participants in hospitals to RCTs in future trials could be considered and systematically assessed. Although it has been argued that work-family specific interventions might help to prevent WFC and its detrimental consequences [67,69,93], hospital workers face the additional difficulty of being able to attend such trainings. Considering WFC in hospitals as a priority becomes even more critical considering this preliminary result. To adapt the treatment to the daily hospital routine and the current personnel situation in German hospitals, the workshop should be shortened to one day. This could be done by sharpening the theoretical contents and the yoga practice.

The maintenance of traditional gender roles in German society, where mothers remain as the primary caregivers of children, even in dual-employment marriages [95], is in line with the participation of a large proportion of women (85.7%). According to evidence that the quality of care provided to children was more strongly related to mothers’ work-family experiences [44,96], efforts to enhance how women manage their work and family responsibilities may have benefits for both the parents and the child. However, to enable fathers to buffer the negative influences of work on family members more effectively [44], strategies to encourage men to reflect upon the subject need to be addressed. Perspectives for future research might be a deeper exploration of gender differences on this topic.

Our results indicate that the group-treatment has the potential to boost self-efficacy: it led to a substantial pre-post increase of self-efficacy. This effect was sustained during the two-month-follow-up, with a large effect size. The intervention did not have a significant effect on children’s health-related quality of life, which was slightly increasing over time in the IG in contrast to the CG. After having risen to some extent between baseline and follow-up, the work-life balance decreased considerably in the IG as compared with the CG between baseline and two-month-follow-up.

To make sense of this pattern of preliminary findings and to address lessons for future research and practice, we can distinguish three types of outcomes: (1) self-efficacy as a personal resource; (2) work-life balance as a predictor of health, family well-being and organizational outcomes and (3) the children’s health-related quality of life as a consequence of parents’ negative work experiences [42,44,45].

Self-efficacy refers to beliefs in one’s capabilities to meet situational demands and successfully carry out a given course of action. Strengthened occupational and family self-efficacy indicates that people feel more competent about managing competing work and family demands. Our results are consistent with other intervention research [66,68,97]. Ten Brummelhuis and Bakker [60] proposed that key resources like self-efficacy, optimism and self-esteem facilitated efficient and effective coping with contextual demands. Self-efficacy, optimism, hope, and resiliency were found to have a significant positive relationship with participants’ work well-being and work-life balance five months later [98]. Future studies could address its mediating role for work-life balance and other health-related and organizational outcomes. Its interactions with job control need to be addressed [99]. Therefore, a longer observation period would be beneficial.

The unexpected reduction of work-life balance between baseline and two-month-follow-up in the IG as compared with the CG may be due to a sensitization process described as a side effect in psychotherapy [100]. According to Grawe [101], by actuation of the problem that motivated a patient to undergo therapy or in this case to take part in the workshop, the problems are made accessible to therapeutic processing. Talking about work-life balance may make participants realize the extent to which they face difficulties in combining work and family roles.

Vahedi and colleagues [43] reported that both work-family conflict and inter-parental conflict represented two potential levers for interventions to produce health benefits for the whole family system. As individuals within families continually influence one another [44,96,102], efforts to reduce stress and strain on employees and to promote work-life balance across generations could include partners as part of these interventions. Another reason why the children’s health-related quality of life did not change might be that the questionnaire was insensitive to our study changes. Due to the unexpectedly small sample size, the KIDSSCREEN-10 could not be examined in detail. In future trials, more specific instruments adapted to age groups or even direct child and adolescent questioning are recommended.

The results might be additionally explained by the fact that as a single-stage intervention, it may not have been significant enough to affect changes in the domains of work-life balance and the children’s health-related quality of life. Indirect support for this assertion can be drawn from the fact that previous interventions to reduce stress at the work-private interface and dysfunctional parenting consisted of a larger number of sessions [65,66,68]. Future research should investigate whether additional booster sessions may lead to more sustained health-related outcomes. However, these should be adaptable to the daily hospital routine. Furthermore, it would be interesting to address the fit between employees and the intervention by considering that persons more vulnerable to WFC benefited more from an initiative which targeted work practices, interactions, and expectation [103].

A limitation of the study might be its small sample size. According to recommendations for reporting results of pilot studies [104], results from hypothesis testing are treated as preliminary and need to be interpreted with caution. Although there were no differences in observed variables at T0 between the IG and the CG, the further threat to internal validity due to the quasi-experimental trial’s nature needs to be taken into consideration, for example in terms of self-selection bias [73]. Competing influences like structural changes in the hospital of interest may have influenced our results. Furthermore, group dynamics, for example influenced by belonging and power, and group-specific impact factors, for example vicarious learning and interpersonal feedback, vary depending on the respective group despite of an active structuring procedure and might therefore have an influence on the effects of the group-treatment. The selection and forming of the group might be considered [75] as well. The reasons for barriers in the recruitment of hospital workers and the loss to follow-up were not assessed systematically, however, the given hospital structures and participants’ professional obligations were the main determinants in this respect, pointing to the urgently needed change at the structural and political level. In addition, the follow-up assessments directly after the intervention and two months later may limit our ability to measure long-term effects. The fact that the KIDSSCREEN-10 could not be examined according to age groups and did only refer to one child per respondent might be another limitation of the study, restricting interpretability of this instrument. The rating of the children’s health-related quality of life in the proxy measure for parents entails potential bias [86]. Although parental proxy reports might be considered as a potential substitute for self-reported ratings [105,106], self-reports and proxy reports both constitute important complementary information concerning children’s health [107]. Furthermore, a social desirability bias in rating scales cannot be excluded. Future studies might use multiple data sources, especially concerning the KIDSSCREEN-10. Moreover, it is difficult to capture the changes reported by participants with quantitative data [108]. Additionally, any bias due to the circumstances when filling in the questionnaire, for example the influence of other persons at home which could not be controlled for or presence of other participants after the group-treatment needs to be taken into consideration. Furthermore, coping with the work-family conflict is not only of high relevance for working parents but also for those with family members in need of special care [38,39]. When the contents for future group-treatments are planned, this could be taken into consideration as an additional subject.

In general, prevention in the healthcare sector is often focused on the individual perspective [109]. However, findings suggest that a nurse’s capacity to cope with work and personal stress is enhanced not only through positive affirmation but also through strong social and collegial support and an infrastructure that supports the provision of quality nursing care [93]. While there is evidence for the success of interventions for promotion of mental health [70], these often individualize feelings and troubles rather than politicizing them. The concept of ‘therapy culture’ highlights the blaming of the individual for structural injustices instead of addressing the origins of these injustices [110,111]. Therefore, it needs to be critically discussed which scope of action healthcare professionals really have in view of large structural difficulties [13,55,112], and if interventions that aim to build individual resilience in the workforce are sufficient to buffer adverse effects. To address these questions, the perspectives for future research would be a study with a larger sample size within a longer observation period.

Research highlights the role of tackling job demands instead of job resources in order to reduce negative health- and job-related outcomes [52]. As dispositions such as negative and positive affect, neuroticism and self-efficacy play the role of antecedents in predicting perceived work and family stressors and WFC [56,113], structural changes on the organizational level could go hand in hand with individual training [7]. The short- and long-term effects of an intervention study targeting the changes undertaken by the hospital to reduce adverse psychosocial work factors show the importance of highlighting the organizational level [114,115]. According to research conducted by Stordeur and colleagues [116], hospitals could work on their attractiveness as employers through the improvement of workplace well-being, for example social support from supervisors and flexible work arrangements. Interventions designed to promote employees’ control over the timing, and to train supervisors to provide personal and performance support to the employees could be useful in reducing the amount of WFC [103]. Gündel and colleagues [55], as well as a study conducted by Lunau and colleagues [36], highlighted not only the individual and the organizational, but also the public health impact on work-life balance, for example working time regulations, appreciation of healthcare workers’ professional efforts and their burden on family life.

The goal on the individual, organizational and political level of intervention could be that instead of conceptualizing the work and family domains as conflicting, work-family spillover could also transfer positive experiences, moods and attitudes between work and family [117] as a benefit for both employers and employees as well as their children.

## 5. Conclusions

Taken together, the results indicate that the group-treatment adapted to the needs of healthcare professionals has the potential to boost self-efficacy in the daily struggle of combining work and family life. The study provides first evidence to the hypothesis that the group-treatment may be valuable for healthcare professionals. However, it does not tackle the structural source of work-family conflict in the healthcare sector, which needs to be addressed by policymakers when it comes to improving working conditions. Perspectives for future research would include a larger study aiming at investigating the effects of a treatment at the individual, organizational and political levels on multiple health- and organization-related outcomes which could empower the mental health of healthcare professionals. Multiple data sources as well as booster sessions might be useful. However, limited resources in the healthcare sector should be addressed.

## Figures and Tables

**Table 1:** Demographic, employment, family, and target variables at baseline for all included participants and divided into group.

	Intervention (*n* = 7) Mean (*SD*)	Control (*n* = 16) Mean (*SD*)	*t* (*df*); *p*	*z*; *p*
Age (years)	38.71 (6.99)	42.25 (10.59)	(21) = −0.80; 0.430 (*ns*)	
Number of children	2.14 (0.69)	1.88 (0.96)		−0.89; 0.413 (*ns*)
Number of children living in joint household	2.14 (0.69)	1.53 (0.92)		−1.62; 0.142 (*ns*)
Mean age of children (years)	7.67 (4.11)	10.19 (8.40)	(20.53) = −0.97; 0.346 (*ns*)	
AGSE ^1^	28.33 (2.07)	30.13 (3.72)	(20) = −1.11; 0.282 (*ns*)	
TS-WLB ^2^	4.43 (0.43)	3.87 (1.01)	(20) = 1.30; 0.208 (*ns*)	
KIDSSCREEN-10 ^3^	42.17 (3.71)	41.64 (4.99)		−0.04; 0.968 (*ns*)
	**%**	**%**	**χ^2^ (*df*); *p***	
Gender (female)	85.7	87.5	(1) = 0.01; 0.907 (*ns*)	
In a solid partnership (yes)	100	87.5	(1) = 0.96; 0.328 (*ns*)	
Employment			(2) = 0.07; 0.964 (*ns*)	
Full-time contract	14.3	18.8		
Parttime contract	71.4	68.8		
Maternal leave	14.3	12.5		
Professional function			(3) = 2.30; 0.512 (*ns*)	
Nursing service	85.7	68.8		
Medical service	14.3	6.3		
Function service in hospital	0.0	6.3		
Other function	0.0	18.8		
Shift working (yes)	100.0	68.8	(1) = 2.80; 0.095 (*ns*)	
Care of relatives with special needs (yes)	28.6	43.8	(1) = 0.47; 0.493 (*ns*)	
Employment of the partner			(2) = 0.96; 0.619 (*ns*)	
Full-time contract	100	87.5		
Parttime contract		6.3		
Other		6.3		

^1^ Adapted General Self-Efficacy Scale [82]. ^2^ Trierer Scale to measure Work-Life Balance [84]. ^3^ Children’s health related quality of life in the proxy measure for parents [86]. *SD* = standard deviation; *df* = degrees of freedom; *ns* = not significant.

**Table 2:** Descriptive statistics and correlations of target variables.

	Mean	*SD*	1	2	3	4	5	6	7	8	9
1. Self-efficacy ^1^ (baseline)	29.64	3.40	(0.78)								
2. Self-efficacy ^1^ (follow-up)	30.79	3.21	0.62 **	(0.76)							
3. Self-efficacy ^1^ (2-month-follow-up)	30.00	4.94	0.43	0.76 **	(0.92)						
4. Work-life-balance ^2^ (baseline)	4.03	0.92	0.35	0.55 *	0.79 **	(0.86)					
5. Work-life-balance ^2^ (follow-up)	3.87	1.02	0.38	0.60 **	0.71 **	0.90 **	(0.86)				
6. Work-life-balance ^2^ (2-month-follow-up)	3.91	0.90	0.33	0.22	0.52 *	0.66 **	0.46	(0.78)			
7. Perceived children’s subjective health and well-being ^3^ (baseline)	41.80	4.55	−0.08	0.15	0.33	0.51 *	0.45	0.56 *	(0.84)		
8. Perceived children’s subjective health and well-being ^3^ (follow-up)	39.22	5.21	−0.23	0.38	0.51 *	0.48	0.41	0.29	0.74 **	(0.87)	
9. Perceived children’s subjective health and well-being ^3^ (2-month-follow-up)	42.32	5.28	−0.12	0.19	0.48 *	0.50 *	0.21	0.48	0.80 **	0.77 **	(0.92)

^1^ [82].^2^ [84].^3^ [86]. *SD* = standard deviation; *N* = 17–22. Cronbach’s alpha in parenthesis (*N* = 12–21). * *p* < 0.05. ** *p* < 0.01.

**Table 3:** Intervention effects: ANOVA.

				Effect on Change Scores (ANOVA ^1^)								
		PP ^2^ Intervention	Control	Group Effect			Time Effect (T0, T1, T2)			Group × Time Effect (T0, T1, T2)		
Outcomes	Time	Mean (*SD*)	Mean (*SD*)	*F*	ɳ_p_^2^	*p*	*F*	ɳ_p_^2^	*p*	*F*	ɳ_p_^2^	*p*
AGSE ^3^	T0	28.40 (2.30)	30.00 (4.07)	0.65	0.04	0.434	2.76	0.17	0.081	5.29	0.27	**0.011**
	T1	32.20 (2.77)	30.55 (3.64)									
	T2	32.60 (3.21)	28.18 (5.17)									
TS-WLB ^4^	T0	4.36 (0.43)	3.89 (1.03)	0.41	0.03	0.531	2.86	0.17	0.074	8.53	0.38	**0.001**
	T1	4.48 (0.41)	3.64 (1.09)									
	T2	3.56 (0.70)	3.98 (0.99)									
KIDSSCREEN-10 ^5^	T0	42.00 (4.12)	42.00 (3.94)	0.32	0.03	0.584	2.84	0.19	0.078	1.40	0.10	0.267
	T1	42.60 (3.91)	39.67 (5.10)									
	T2	43.80 (4.15)	42.67 (5.74)									

^1^ Analysis of variance. ^2^ Per protocol analysis. ^3^ Adapted General Self-Efficacy Scale [82]. ^4^ Trierer Scale to measure Work-Life Balance [84]. ^5^ Children’s health related quality of life in the proxy measure for parents [86]. AGSE: *n*IG = 5, *n*CG = 11, TS-WLB: *n*IG = 5, *n*CG = 11, KIDSSCREEN-10: *n*IG = 5, *n*CG = 9. T0-T2 measurement points at baseline, directly after the treatment and two months later. *SD* = standard deviation. Boldface indicates *p* < 0.05. Two-tailed significance.

**Table 4:** Intervention effects: Contrasts.

	Pre-Post Change							
	Group Difference in Pre-Post Change (T1-T0)				Group Difference in Pre-Post Change (T2-T0)			
Outcomes	Mean (95% CI)	*F*	*r*	*p*	Mean (95% CI)	*F*	*r*	*p*
AGSE ^1^	3.25 (0.08–6.43)	4.83	0.51	**0.045**	6.02 (1.03–11.01)	6.69	0.57	**0.022**
TS-WLB ^2^	0.37 (−0.13–0.88)	2.51	0.39	0.136	−0.89 (−1.64–(−0.14))	6.42	0.56	**0.024**
KIDSSCREEN-10 ^3^	2.93 (−0.90–6.76)	2.78	0.43	0.121	1.13 (−2.35–4.62)	0.50	0.20	0.492

^1^ Adapted General Self-Efficacy Scale [82]. ^2^ Trierer Scale to measure Work-Life Balance [84]. ^3^ Children’s health related quality of life in the proxy measure for parents [86]. AGSE: *n*IG = 5, *n*CG = 11, TS-WLB: *n*IG = 5, *n*CG = 11, KIDSSCREEN-10: *n*IG = 5, *n*CG = 9. T0-T2 measurement points at baseline, directly after the treatment and two months later. Positive group differences indicate an increase in the respective outcome in the intervention as compared to the control condition, and vice versa for negative estimates. CI = confidence interval. Boldface indicates *p* < 0.05. Two-tailed significance.

## Data Availability

The data sets generated for this study will not be made publicly available due to privacy reasons. The data supporting the results of this study can be requested from NH, but restrictions apply if the use could endanger the anonymity of the participants.

## Appendix A

**Table A1:** Skala zur Erfassung der adaptierten Selbstwirksamkeitserwartung [Adapted General Self-Efficacy Scale].

#	Item
1	Ich finde Mittel und Wege, Beruf und Familie trotz Widerständen zu vereinbaren. [I find ways and means to reconcile work and family despite obstacles.]
2	Die Vereinbarkeit von Beruf und Familie gelingt mir immer, wenn ich mich darum bemühe. [I always succeed in balancing work and family life if I make an effort to do so.]
3	Es bereitet mir keine Schwierigkeiten, meine beruflichen und familiären Ziele/Pflichten zu vereinbaren. [I have no difficulty balancing my professional and family goals/duties.]
4	In unerwarteten Situationen weiß ich immer, wie ich mich verhalten soll. [In unexpected situations, I always know how to act.]
5	Auch bei überraschenden Ereignissen im Beruf oder in der Familie glaube ich, dass ich damit gut zurechtkommen kann. [I believe that I can cope well even with surprising events at work or in the family.]
6	Schwierigkeiten im Beruf oder in der Familie sehe ich gelassen entgegen, weil ich meinen Fähigkeiten immer vertrauen kann. [I face difficulties at work or in my family calmly because I can always trust my abilities.]
7	Was auch immer passiert, ich werde schon klarkommen. [Whatever happens, I will manage it.]
8	Für jedes Problem, welches meinen Beruf oder meine Familie betrifft, kann ich eine Lösung finden. [For every problem concerning my job or my family, I can find a solution.]
9	Wenn Veränderungen im Beruf oder in der Familie auf mich zukommen, weiß ich, wie ich damit umgehen kann. [When changes come my way at work or in my family, I know how to deal with them.]
10	Wenn ein Problem auftaucht, kann ich es aus eigener Kraft meistern. [When a problem arises, I can cope with it on my own.]
